# DNMT1-induced miR-378a-3p silencing promotes angiogenesis via the NF-κB signaling pathway by targeting TRAF1 in hepatocellular carcinoma

**DOI:** 10.1186/s13046-021-02110-6

**Published:** 2021-11-08

**Authors:** Bin Zhu, Jun-Jie Chen, Ying Feng, Jun-Ling Yang, Hua Huang, Wen Yuan Chung, Yi-Lin Hu, Wan-Jiang Xue

**Affiliations:** 1 Department of General Surgery, Affiliated Hospital of Nantong University, 20 Xisi Street, Nantong, 226001 Jiangsu China; 2 Research Center of Clinical Medicine, Affiliated Hospital of Nantong University, 20 Xisi Street, Nantong, 226001 Jiangsu China; 3 Medical school, Nantong University, 19 Qixiu Road, Nantong, 226001 Jiangsu China; 4 Department of Pathology, Affiliated Hospital of Nantong University, 20 Xisi Street, Nantong, 226001 Jiangsu China; 5 Department of Hepatobiliary and Pancreatic Surgery, Leicester General Hospital, University of Leicester, Gwendolen Road, Leicester, LE5 4PW UK

**Keywords:** Angiogenesis, HCC, miR-378a-3p, TRAF1, DNA methylation

## Abstract

**Background:**

Angiogenesis plays an important role in the occurrence, development and metastasis of hepatocellular carcinoma (HCC). According to previous studies, miR-378a participates in tumorigenesis and tumor metastasis, but its exact role in HCC angiogenesis remains poorly understood.

**Methods:**

qRT-PCR was used to investigate the expression of miR-378a-3p in HCC tissues and cell lines. The effects of miR-378a-3p on HCC in vitro and in vivo were examined by Cell Counting Kit-8 (CCK-8), Transwell, tube formation and Matrigel plug assays, RNA sequencing, bioinformatics, luciferase reporter, immunofluorescence and chromatin immunoprecipitation (ChIP) assays were used to detect the molecular mechanism by which miR-378a-3p inhibits angiogenesis.

**Results:**

We confirmed that miR-378a-3p expression was significantly downregulated and associated with higher microvascular density (MVD) in HCC; miR-378a-3p downregulation indicated a short survival time in HCC patients. miR-378a-3p knockdown led to a significant increase in angiogenesis in vitro and in vivo. We found that miR-378a-3p directly targeted TNF receptor associated factor 1 (TRAF1) to attenuate NF-κB signaling, and then downregulated secreted vascular endothelial growth factor. DNA methyltransferase 1 (DNMT1)-mediated hypermethylation of miR-378a-3p was responsible for downregulating miR-378a-3p. Moreover, a series of investigations indicated that p65 initiated a positive feedback loop that could upregulate DNMT1 to promote hypermethylation of the miR-378a-3p promoter.

**Conclusion:**

Our study indicates a novel DNMT1/miR-378a-3p/TRAF1/NF-κB positive feedback loop in HCC cells, which may become a potential therapeutic target for HCC.

**Supplementary Information:**

The online version contains supplementary material available at 10.1186/s13046-021-02110-6.

## Background

Hepatocellular carcinoma (HCC) is one of the most common digestive tract tumors in the world, with approximately 850,000 new cases and at least 780,000 related deaths annually [1]. With advancements in diagnostic techniques and the development of surgical strategies, such as hepatectomy, liver transplantation, local ablation therapy, and transarterial chemoembolization, the survival rate and quality of life of HCC patients have significantly improved; however, the five-year survival rate is still less than 30% [2]. Angiogenesis is known to regulate tumor growth and metastasis by providing nutrients for tumor cells [3]. HCC is a typical angio-rich tumor characterized by abnormal angiogenesis. In clinical practice, multi-target tyrosine kinase inhibitors targeting vascular endothelial growth factor (VEGF) could inhibit the growth and development of HCC by suppressing angiogenesis. However, these drugs still have limitations [4–6]. Therefore, it is vital to explore new targets for the treatment of HCC by regulating HCC angiogenesis.

NF-κB pathway is a highly conserved signaling pathway, and its abnormal activation plays an important role in tumor occurrence and development. It is known that NF-κB has a wide range of transcriptional activities and multiple links to angiogenesis [7, 8]. It has been proved that inhibition of NF-κB could eliminate the production of VEGF and then induce the suppression of angiogenesis in a variety of types of cancer [9]. Studies have indicated that NF-κB contributes to angiogenesis in prostate [10], breast [11], colorectal [12], and pancreatic cancers [13]. Inhibition of meiotic recombination protein (REC8) has been shown to promote NF-κB/p65 activity and its downstream gene VEGF, leading to tumor angiogenesis in the gastric cancer [14]. The expression of prenyl diphosphate synthase subunit 2 (PDSS2-DEL2) was found to be positively related to activation of the NF-κB pathway, leading to the metastasis and angiogenesis of HCC [15]. Therefore, inhibiting NF-κB activity, leading to decreased tumor-induced blood vessel formation, might be an important strategy for the treatment of HCC.

MicroRNAs (miRNAs) are endogenous noncoding RNAs, that are approximately 22 nucleotides in length, and are known to regulate various biological processes, including angiogenesis and tumor progression [16, 17]. miRNA-378a, including miR-378a-3p and miR-378a-5p, is located on human chromosome 5q32 and has abundant biological functions [18]. The expression of miR-378a is involved in the regulation of mitochondria, glucose metabolism, autophagy, and other metabolic pathways [19, 20]. In addition, miR-378a plays an active role in the occurrence and development of malignant tumor [21, 22]. miR-378a-5p suppresses angiogenesis in oral squamous cell carcinoma by regulating of Kallikrein-related peptidase 4 (KLK4). miR-378a-3p could bind to the target genes, mitogen-activated protein kinase 1 (MAPK1) and growth factor receptor bound protein 2 (GRB2), leading to the silencing of their expression and reversing the cisplatin resistance of ovarian cancer cells [23]. The overexpression of miR-378 also promotes the migration and invasion of human hepatoblastoma cells [24]. Pogribny et al. fed rats with the oncogenic agent tamoxifen to induce HCC. After 12 weeks and 24 weeks, the miRNA expression profiles were studied in the livers of rats, and it was found that miR-378 expression was decreased in the tamoxifen-treated group versus the control group [25]. However, the mechanism by which miR-378a affects the occurrence and development of HCC is still unclear.

In this study, we identified a novel function of miR-378a-3p: it plays an antitumor role in HCC angiogenesis, and high miR-378a-3p expression is correlated with a favorable prognosis. Further in vitro and in vivo experiments demonstrated that miR-378a-3p abolished the oncogenic function of NF-κB/p65 to silence its targeted gene TNF receptor associated factor 1 (TRAF1), an activator of NF-κB pathway. Moreover, p65 promoted the transcriptional expression of DNA methyltransferase 1 (DNMT1), which could promote miR-378a-3p promoter methylation. Overall, the regulatory network of HCC angiogenesis forms a positive feedback loop via DNMT1/miR-378a-3p/TRAF1/NF-κB.

## Materials and methods

### Clinical samples

A total of 108 pairs of primary HCC tissues and paracarcinoma tissues were obtained from patients undergoing surgery at the affiliated hospital of Nantong University between 2004 and 2010. Another cohort of 10 matched fresh HCC samples were collected from the same hospital. None of the patients had received chemotherapy, radiotherapy, or immunotherapy before undergoing surgery. The follow-up was completed in August 2015 (median follow-up 65 months; range, 2–95 months). The samples were collected promptly during surgical resection and stored at − 80 °C. The experimental protocol was approved by the ethics committee of the Affiliated Hospital of Nantong University. Additionally, written informed consent was obtained from all participants.

### Cell lines and culture condition

The MHCC-97H, MHCC-97 L and HCCLM3 cell lines were donated by the Liver Cancer Institute, ZhongShan Hospital. LO2, SMMC-7721 and human umbilical vein endothelial cells (HUVECs) were procured from GeneChem (Shanghai, China). All cell lines were cultured in Dulbecco’s Modified Eagle’s Medium (DMEM, Invitrogen, USA), supplemented with 10% fetal bovine serum (FBS, Clark Bioscience, USA), ampicillin (Gibco, USA), and streptomycin (Gibco, USA) in a 5% CO_2_ humidified chamber at 37 °C.

### Cell transfection and drug treatment

The sequences of the mimic or inhibitors were designed and composed by GenePharma (Suzhou, China). The miR-378a-3p agomir and agomir-NC were synthesized and purified by GenePharma (Suzhou, China). Cell transfection was performed on a six-well plate using Lipofectamine 3000 reagent (Invitrogen, USA). Cells were collected 48 h post-transfection to detect the mRNA expression. DNMT1 siRNA, DNMT3A siRNA, DNMT3B siRNA, and TRAF1 siRNA were obtained from GenePharma (Suzhou, China). TRAF1, p65 and DNMT1 overexpression plasmid were obtained from GeneChem (Shanghai, China). The siRNA sequences are shown in Table S1 [26, 27]. For drug treatment, cells were treated with 10 μM 5-azacytidine (5-Aza, MedChemExpress, Shanghai, China) for 48 h, 50 μg/ml SN50 (MedChemExpress, New Jersey, USA) for 24 h, or 60 μg/ml lipopolysaccharide (LPS, Solarbio, Beijing, China) for 24 h. All experiments were performed in triplicate.

### qRT-PCR analysis and fluorescence in situ hybridization (FISH) assay

Total RNA was extracted with TRIzol reagent (Invitrogen, USA). qRT-PCR was performed following a previously described method [28]. The bulge-loop RT primer and qPCR primers specific for hsa-miR-378a-3p and hsa-miR-378a-5p were designed and synthesized by RiboBio (Guangzhou, China). Table S2 lists the primers used in this study. A miR-378a-3p FISH kit was purchased from GenePharma (Suzhou, China) and the experiment was performed according to the manufacturer’s instructions [29]. The results were analyzed by an IX71 inverted microscope (Olympus, Japan). All experiments were performed in triplicate.

### Subcellular fractionation and western blot assay

Subcellular fractionation was performed with a Nuclear and Cytoplasmic Protein Extraction Kit (Sangon, Shanghai, China), in accordance with the manufacturer’s instructions. Total protein separation and western blotting were performed as described previously [30]. The following antibodies were used: anti-p-IκBα (Cell Signaling Technology, USA), anti-p-IKKβ (Abcam, Cambridge, MA, USA), anti-β-actin, anti-Lamin B1, anti-TRAF1 and anti-NF-κB p65 (Proteintech, Wuhan, China). All experiments were performed in triplicate.

### Immunohistochemistry (IHC)

IHC was performed following a previously described method [30]. The following antibodies were used: anti-CD34, anti-VEGF, anti-DNMT1, anti-TRAF1, and anti-NF-κB p65 (Proteintech, Wuhan, China). Staining intensity was scored manually by two independent experienced pathologists as follows: 0 = no staining, 1 = weak staining, 2 = moderate staining, and 3 = strong staining. The percentage of positive cells was also assessed according to four scores: 1 (0-10%), 2 (11-50%), 3 (51-80%), and 4 (81-100%). The final IHC score was calculated by multiplying the intensity score by the percentage of positive cells. CD34 antibody was used to stain vascular endothelial cells, and then the microvessel density (MVD) was calculated. The field of maximal CD34 expression was found in tumor tissues. Within this field, the area of maximal angiogenesis was selected, and microvessels were counted at 200× magnification. Low MVD was indicated by scores from 0 to 3. High MVD was indicated by scores ≥4 [31].

### Immunofluorescence assays

Immunofluorescence assays were performed as described in our previous study [30]. Treated HCC cells were washed three times with cold phosphate-buffered saline (PBS, Gibco, USA), fixed with 4% paraformaldehyde (Beyotime, Shanghai, China) for 20 min, treated with 0.1% of Triton X-100 (Beyotime, Shanghai, China) for 5 min and blocked in 5% Bovine Serum Albumin (Solarbio, Beijing, China). Then cells were incubated overnight with anti-NF-κB p65 (Proteintech, Wuhan, China) at 4 °C, followed by washing three times with PBS. Next, the cells were incubated with fluorescent Alexa Fluor 594-conjugated goat anti-rabbit IgG (ABclonal, Wuhan, China). Finally, the nuclei were labelled with DAPI (Cell Signaling Technology, USA) for 15 min, and the images of stained cells were captured by the BX41 microscope (Olympus, Japan). All experiments were performed in triplicate.

### Wound healing, cell invasion and colony formation assay

We added 0.4-μm-thick pore inserts (Corning, USA) to 6-well culture plates. HUVECs (1 × 10^6^ cells) were placed in the lower chambers and treated HCCLM3 and SMMC-7721 cells were placed in the upper chambers and co-cultured in DMEM with 5% FBS for 48 h. Next, we collected the treated HUVECs for wound healing assays, cell invasion assays, and colony formation assays. These experiments were performed as described previously [32]. All experiments were performed in triplicate.

### Tube formation assay

We added Matrigel (170 μL, BD Biosciences, USA) to cold 48-well culture plates and allowed it to solidify at 37 °C for 30 min. Next, treated HUVECs (1 × 10^4^ cells/well) were seeded onto the Matrigel layer. After incubation for 8 h at 37 °C, the formation of polygonal tubes was assessed microscopically at 100x magnification. All experiments were performed in triplicate.

### Experiments with animals

Male BALB/C nude mice aged 6 weeks were purchased from the Animal Laboratory Center of Nantong University (Nantong, China). The matrix plug assay was used to assess angiogenic capacity. HCCLM3 and SMMC-7721 cells were treated with miR-378a-3p mimic or inhibitors for 48 h. After that, the cells were resuspended in a mixture of serum-free medium and Matrigel (BD Biosciences, USA) at a density of 5 × 10^6^ cells/450 μL and subcutaneously injected into nude mice (5 mice for each group). The nude mice were euthanized 7 days later, and the matrix plug was removed for further analysis. The subcutaneous xenograft mouse model was used to assess the tumor growth rate. HCCLM3 cells (5 × 10^6^) transfected with miR-378a-3p agomir or agomir-NC for 48 h, were subcutaneously injected into the upper part of the forelimb (5 mice per group). Tumor volume was measured every three days and calculated as V = 0.5 × length×width^2^. The nude mice were sacrificed after 24 days, and the tumor tissues were extracted and collected for subsequent studies [33]. All animal experiments were approved by the Institutional Animal Care and Use Committee of Nantong University following the current guidelines for animal care and welfare.

### Elisa

HCC cells were seeded in 6-well plates and incubated in serum-free medium for 24 h. The conditioned medium was collected, and the concentration of VEGF was quantified using VEGF ELISA kits (Jianglai Bio, Shanghai, China) according to the manufacturer’s instructions. All experiments were performed in triplicate.

### Luciferase reporter assays

NF-κB luciferase assays were performed via the co-transfection with the NF-κB-luciferase plasmid, control luciferase plasmid, pRL-TK Renilla, miRNA mimic and miRNA mimic control into HCC cells by Lipofectamine 3000. The 3′- untranslated region (UTR) sequences of TRAF1 containing miR-378a-3p binding sites were synthesized and cloned into the luciferase reporter vector. Next, SMMC-7721 and HCCLM3 cells transfected with miRNA mimic and control were co-transfected with luciferase reporter vectors. The wild-type DNMT1 promoter and a promoter with mutated NF-κB-binding sites were designed by GeneChem. DNMT1-WT or DNMT1-MUT was co-transfected with pcDNA3.1 vector or pcDNA3.1 p65. After 48 h, the luciferase activity was measured with a dual-luciferase assay kit (Beyotime, Shanghai, China). The results are presented as the relative luciferase activity of Renilla, which was normalized to the activity of firefly luciferase. All the luciferase reporter assays plasmids were purchased from GeneChem (Shanghai, China). All experiments were performed in triplicate.

### Chromatin immunoprecipitation (ChIP)

ChIP was performed using a previously described method [34]. ChIP assays were performed with a Pierce Magnetic ChIP Kit (Thermo Fisher Scientific, USA) according to the manufacturer’s protocol. Anti-p65 antibody and normal IgG (Multiscience, Hangzhou, China) were used for immunoprecipitation. The primer sequences for the ChIP assays were shown in Table S2. All experiments were performed in triplicate.

### Online bioinformatics analysis

GSE54751, GSE108724 and GSE174608 were downloaded from the Gene Expression Omnibus (GEO) database to assess the differential miR-378a-3p expression. The Cancer Genome Atlas (TCGA) database was used to analyse the expression correlation between miR-378a-3p and DNMT1, TRAF1 or VEGFA, miR-378a expression, and the prognostic significance of DNMT1 and VEGFA. Putative miR-378a-3p target genes were predicted by miRWalk (http://mirwalk.umm.uni-heidelberg.de/) and miRTarBase (http://mirtarbase.mbc.nctu.edu.tw/). The Gene Expression Profiling Interactive Analysis (GEPIA) database (http://gepia2.cancer-pku.cn/) was used to analyse the expression correlation between DNMT1 and p65. JASPAR (jaspar.genereg.net/) was used to predict the putative transcription factors of DNMT1. The Search Tool for the Retrieval of Interacting Genes/Proteins (STRING) dataset (https://string-db.org/) was used to predict the functional pathways correlated with TRAF1. SurvExpress data (http://bioinformatica.mty.itesm.mx:8080/Biomatec/SurvivaX.jsp) was used to predict the prognostic significance of TRAF1.

### RNA sequencing (RNA-seq)

Three repeated pairs of SMMC-7721/miR-378a-3p mimic and control groups were prepared for RNA-seq, which was performed by GENEWIZ (Soochow, Suzhou, China). Significant differences in mRNA expression (*P*-value < 0.05 and |log2 FC| > 1) between groups were identified using the fold change cut-off.

### Statistical analysis

The measured data are presented as the mean ± SD. Student’s t-test was used for statistical comparisons between experimental groups. The correlations between miR-378a-3p expression and various clinicopathological factors were performed using the chi-squared test. The Cox regression model was used to evaluate prognostic factors. Logistic regression analysis was performed to identify risk factors affecting miR-378a-3p levels in HCC. The probability of differences in overall survival (OS) and disease-free survival (DFS) were assessed with Kaplan–Meier and log-rank tests. Spearman’s correlation rank analysis was used to analyze categorical variables. All statistical analyses were performed with SPSS v24.0 software. *P* < 0.05 was considered to indicate a statistically significant difference.

## Results

### miR-378a-3p expression is downregulated in HCC patients and correlated with HCC angiogenesis

**Fig. 1:**
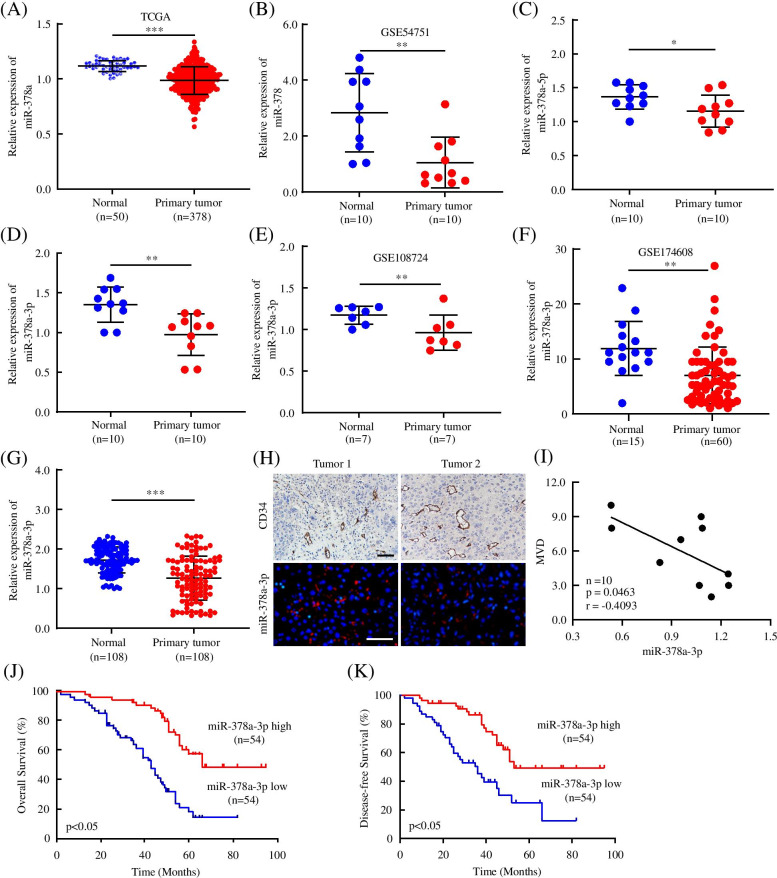
miR-378a-3p expression is downregulated in HCC patients and correlated with HCC angiogenesis. **A, B** The mRNA expression of miR-378a in HCC and paired normal tissues was analyzed based on the TCGA and GEO databases. **C, D** Two chains of miR-378a were detected in 10 matched paired normal tissues and HCC tissues by qRT-PCR. **E-G** Relative miR-378a-3p mRNA expressions was measured in GEO datasets and in 108 pairs of specimens. **H** Tumor sections were stained with anti-CD34 for IHC assays and miR-378a-3p for FISH assays. **I** The correlation line between miR-378a-3p and MVD was analyzed by linear regression analysis. **J, K** OS and DFS were analyzed in HCC patients with high or low expression of miR-378a-3p by the Kaplan–Meier method. *P < 0.05; **P < 0.01; ***P < 0.001

To elucidate the functional roles of miR-378a in HCC, we found that miR-378a levels were downregulated in HCC tissues compared to normal tissues from the TCGA and GSE54751 datasets (Fig. 1A, B). Next, we compared the mRNA levels of the two strands of miR-378a, miR-378a-3p and miR-378a-5p, in 10 matched fresh HCC and corresponding normal tissues. miR-378a-3p and miR-378a-5p expression were significantly downregulated in HCC, and miR-378a-3p showed a substantially higher difference (Fig. 1C, D). Analysis of the GSE108724 and GSE174608 datasets and 108 matched samples also confirmed that miR-378a-3p was downregulated in HCC (Fig. 1E-G). We combined the clinical data of 108 patients with the data for the corresponding samples, and we found that the decreased miR-378a-3p expression was closely associated with thrombosis of tumor blood vessel and MVD (Table 1). Moreover, multivariate logistic regression analysis showed that the expression of miR-378a-3p was significantly inversely correlated with MVD (*p* < 0.001, odds ratio [OR] = 0.070, 95% confidence interval [CI]: 0.027–0.179). The results were also validated in 10 paired HCC and normal tissues via a FISH assay for assessing miR-378a-3p and IHC for assessing MVD (Fig. 1H, I). Kaplan–Meier analysis showed that HCC patients with low miR-378a-3p expression had worse OS and DFS rates than those with higher miR-378a-3p expression (Fig. 1J, K). Additionally, the results of Cox multivariate regression analysis revealed that miR-378a-3p was an independent, effective prognostic factor (Table 2).

**Table 1:** Relationships between miR-378a-3p expression and clinicopathological characteristics of HCC patients

Clinicopathological characteristics	n	Low expression	High expression	***P*** value
**Total**	108	54	54	
**Gender**				0.060
Male	75	33	42	
Female	33	21	12	
**Age (years)**				0.440
≤ 60	83	41	42	
> 60	25	13	12	
**Grade of differentiation**				0.123
Low	56	32	24	
High-Middle	52	22	30	
**Tumor diameter (cm)**				0.123
≤ 5	50	29	21	
> 5	58	25	33	
**Liver function (Child-Pugh stage)**				0.588
A	92	45	47	
B or C	16	9	7	
**Hepatocirrhosis**				0.288
Absent	31	13	18	
Present	77	41	36	
**HBV infection**				0.555
Absent	43	20	23	
Present	65	34	31	
**Thrombosis of tumor blood vessel**				0.011
Absent	65	26	39	
Present	43	28	15	
**AFP (ng/ml)**				0.083
≤ 20	53	22	31	
> 20	55	32	23	
**BCLC stage**				0.552
A	41	19	22	
B, C, or D	67	35	32	
**Envelope**				0.700
Absent	52	25	27	
Present	56	29	27	
**Tumor satellite**				0.079
Absent	63	27	36	
Present	45	27	18	
**MVD**				< 0.001
High	59	45	14	
Low	49	9	40	

*AFP* serum alpha fetoprotein, *HBV* hepatitis B virus, *BCLC* Barcelona Clinic Liver Cancer

**Table 2:** Univariate and multivariable analyses of OS and DFS in HCC patients

Variable	OS			DFS		
	Univariate analysis	Multivariable analysis		Univariate analysis	Multivariable analysis	
	***P*** > |z|	***P*** > |z|	HR(95%CI)	***P*** > |z|	***P*** > |z|	HR(95%CI)
**miR-378a-3p expression**						
Low (*n* = 54) vs. high (*n* = 54)	< 0.001	0.003	0.414(0.232-0.736)	0.001	0.010	0.454(0.249-0.828)
**Gender**						
Male (*n* = 75) vs. female (*n* = 33)	0.944			0.737		
**Age (years)**						
≤ 60 (*n* = 83) vs. > 60 (*n* = 25)	0.518			0.837		
**Grade of differentiation**						
Low (*n* = 56) vs. middle-high (*n* = 52)	0.330			0.444		
**Tumor diameter (cm)**						
≤ 5 (*n* = 50) vs. > 5 (*n* = 58)	0.588			0.294		
**Liver function (Child-Pugh stage)**						
A (*n* = 92) vs. B or C (*n* = 16)	0.901			0.980		
**Hepatocirrhosis**						
Absent (*n* = 31) vs. present (*n* = 77)	0.874			0.698		
**Hepatitis B virus**						
Absent (*n* = 43) vs. present (*n* = 65)	0.189			0.692		
**Tumor thrombus**						
Absent (*n* = 65) vs. present (*n* = 43)	0.079			0.052		
**AFP (ng/ml)**						
≤ 20 (*n* = 53) vs. > 20 (*n* = 55)	0.264			0.973		
**BCLC stage**						
I (*n* = 41) vs. II, III, or IV (*n* = 67)	0.194			0.149		
**Envelope**						
Absent (*n* = 52) vs. present (*n* = 56)	0.142			0.946		
**Tumor satellite**						
Absent (*n* = 63) vs. present (*n* = 45)	0.146			0.411		
**MVD**						
High (*n* = 59) vs. Low (*n* = 49)	< 0.001	0.018	2.048(1.129-3.715)	0.001	0.022	2.036(1.106-3.746)

### miR-378a-3p inhibited HCC angiogenesis in vitro and in vivo

**Fig. 2:**
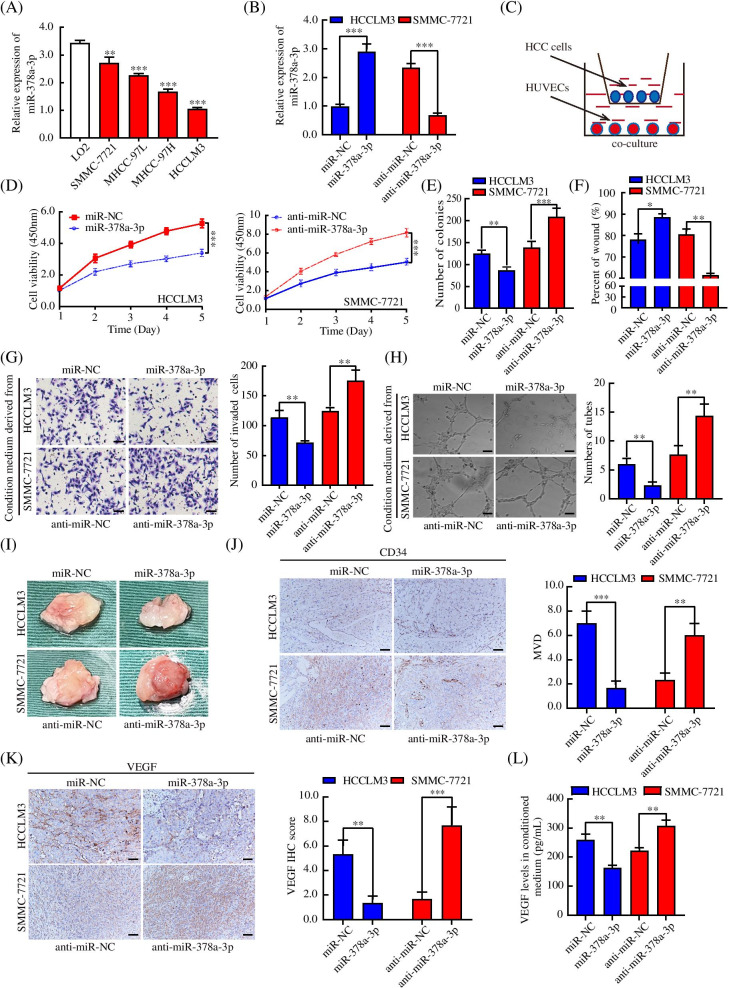
miR-378a-3p expression inhibited HCC angiogenesis in vitro and in vivo. **A** qRT-PCR was used to analyze the mRNA expression of miR-378a-3p in different HCC cell lines. **B** HCCLM3 and SMMC-7721 cells were transfected with miR-378a-3p mimic, mimic-NC, inhibitor, or inhibitor-NC and analyzed by qRT-PCR. **C** A working model of co-culture. **D, E** Treated HUVECs were evaluated by the CCK-8 and colony formation assays to analyze cell viability. **F, G** Wound healing assay and invasion assay were performed, and the numbers of migrating, or invading cells/field were recorded. **H** miR-378a-3p mimic, mimic-NC, inhibitor, or inhibitor-NC RNA was transfected into HCCLM3 and SMMC-7721 cells for 48 h, and then tube formation assays were performed for HUVECs co-cultured with conditioned medium collected from HCC cells. **I** Matrigel plugs with treated HCC cells were used to assess angiogenesis potential**. J, K** Matrigel plugs were collected to perform IHC analysis using anti-CD34 and anti-VEGF antibodies. **L** The VEGF protein concentration in the conditioned medium of treated HCC cells was detected by ELISA. *P < 0.05; **P < 0.01; ***P < 0.001

To further study the function of miR-378a-3p in HCC angiogenesis. qRT-PCR was performed to investigate the expression of miR-378a-3p in four HCC cell lines and the human normal liver cell line LO2. The expression of miR-378a-3p in HCC cell lines was significantly lower than that in LO2 cells (Fig. 2A). Then, we established miR-378a-3p-overexpressing HCCLM3 and MHCC-97H cells and miR-378a-3p-knockdown SMMC-7721 and MHCC-97 L cells (Fig. 2B, Fig. S1A). A co-culture system was used to culture the treated HCC cells along with HUVECs for 48 h (Fig. 2C). Reduced proliferation was observed in HUVECs co-cultured with HCC cells with high miR-378a-3p expression (Fig. 2D, E, Fig. S1B-F). Wound healing assays and Transwell assays indicated that the migration and invasion ability of HUVECs was decreased after co-culture with the miR-378a-3p-overexpressing HCC cells, while miR-378a-3p knockdown increased migration and invasion (Fig. 2F, G, Fig. S1G-K). The supernatant from miR-378a-3p-overexpressing HCC cells inhibited HUVEC tube formation (Fig. 2H, Fig. S1L-M). The Matrigel plug assay was used to analyze the neovascularization potential to further identify the antitumor function of miR-378a-3p in HCC. The Matrigel plugs collected from the miR-NC group had more blood vessels (Fig. 2I). The IHC results showed that the MVD and the expression of VEGF were lower in the miR-378a-3p-overexpression group than in the NC group (Fig. 2J, K). The ELISA results indicated that the secretion of VEGF was downregulated by the miR-378a-3p mimic and upregulated by the miR-378a-3p inhibitor in the conditioned medium of HCC cell lines (Fig. 2L, Fig. S1N). Thus, our data suggested that miR-378a-3p negatively regulated HCC angiogenesis in vitro and in vivo.

### TRAF1 acted as a direct target gene of miR-378a-3p in HCC cells

**Fig. 3:**
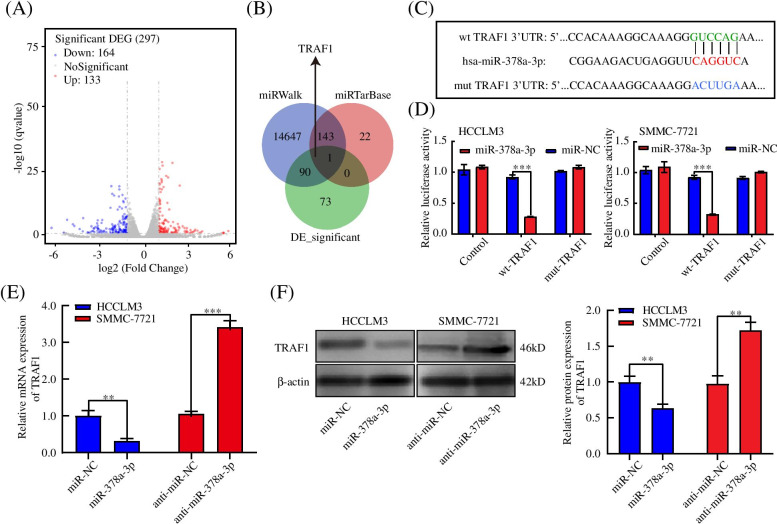
TRAF1 acted as a direct target gene of miR-378a-3p in HCC cells. **A** RNA-seq identified differentially expressed genes between SMMC-7721-overexpressing and NC cells. **B** Venn diagram of the potential target of miR-378a-3p using two independent algorithms (miRWalk and miRTarBase) combined with our RNA-seq. **C** Prediction of the potential binding site of miR-378a-3p and TRAF1 through bioinformatics websites. **D** Relative luciferase activity in HCCLM3 and SMMC-7721 cells co-transfected with miR-378a-3p mimic and WT or MUT TRAF1. **E**, **F** TRAF1 mRNA and protein levels in HCCLM3 and SMMC-7721 cells. *P < 0.05; **P < 0.01; ***P < 0.001

RNA-seq was performed to understand the molecular mechanism of miR-378a-3p-induced inhibition of angiogenesis in SMMC-7721 cells. We identified 297 differentially expressed genes (DEGs), including 133 upregulated DEGs and 164 downregulated DEGs (Fig. 3A). Next, we used the online bioinformatics tools miRWalk and miRTarBase to predict the potential target of miR-378a-3p. Combined with these data, TRAF1 was seen as the only putative target of miR-378a-3p for further research (Fig. 3B). According to the software prediction, we found that TRAF1 had a potential binding site of miR-378a-3p, and then we inserted wild-type or mutant TRAF1 into a luciferase reporter vector (Fig. 3C). Transfection of the miR-378a-3p mimic significantly inhibited the luciferase activity of the wild-type (WT) TRAF1 reporter, and this effect was completely eradicated for the mutant-type (MUT) reporter (Fig. 3D). Additionally, the miR-378a-3p mimic downregulated the mRNA and protein expression of TRAF1, while the miR-378a-3p inhibitor upregulated TRAF1 expression in HCC cells (Fig. 3E, F). Thus, these results indicated that TRAF1 acted as a direct target of miR-378a-3p in HCC.

### miR-378a-3p inhibited HCC angiogenesis by targeting TRAF1

**Fig. 4:**
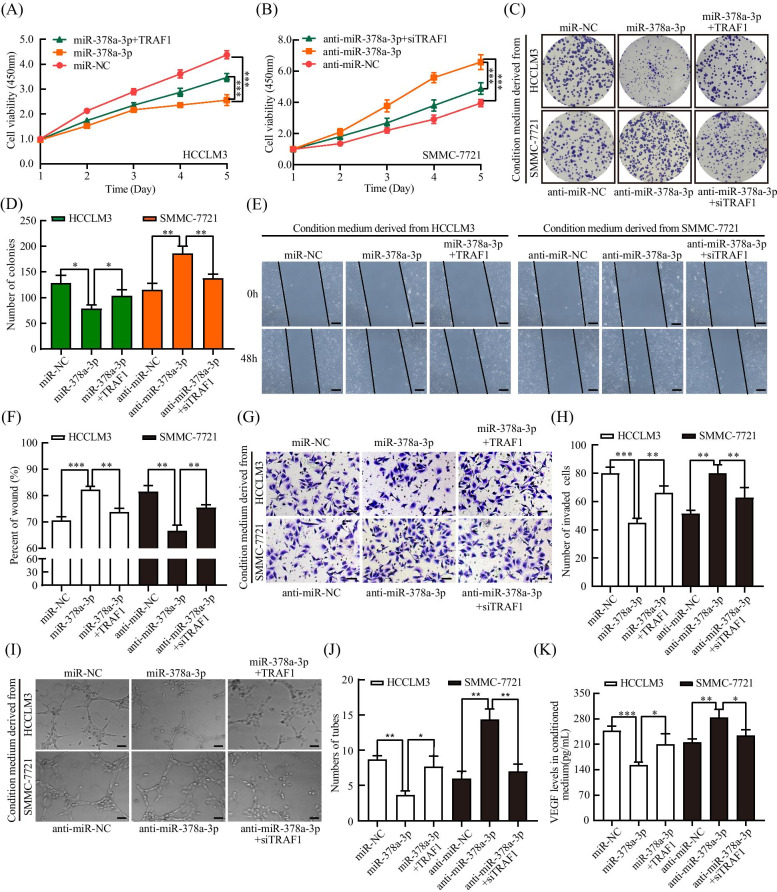
miR-378a-3p inhibited HCC angiogenesis by targeting TRAF1. **A-D** Cell viability of HCCLM3 and SMMC-7721 cells transfected with mimic NC, miR-378a-3p, or miR-378a-3p plus TRAF1 and anti-miR-NC, anti-miR-378a-3p, or anti-miR-378a-3p plus siTRAF1 detected by CCK-8 and colony formation assays. **E-J** Migration, invasion and angiogenesis of HCCLM3 and SMMC-7721 cells transfected with mimic NC, miR-378a-3p, or miR-378a-3p plus TRAF1 and anti-miR-NC, anti-miR-378a-3p, or anti-miR-378a-3p plus siTRAF1 detected by wound healing, invasion and tube formation assays. **K** VEGF protein concentration in the condition medium of HCCLM3 and SMMC-7721 cells transfected with mimic NC, miR-378a-3p, or miR-378a-3p plus TRAF1 and anti-miR-NC, anti-miR-378a-3p, or anti-miR-378a-3p plus siTRAF1, as detected by ELISA. *P < 0.05; **P < 0.01; ***P < 0.001

Rescue experiments were performed to further confirm whether silencing miR-378a-3p could facilitate HCC angiogenesis by upregulating TRAF1 expression. The analysis showed that, compared with transfection of miR-378a-3p mimic alone, simultaneous transfection of TRAF1-overexpressing plasmid could reverse the decrease in angiogenesis and secreted VEGF levels in conditioned medium, while the cells transfected with miR-378a-3p inhibitor and TRAF1 siRNA exhibited significantly lower angiogenesis ability and levels of secreted VEGF than the cells transfected with miR-378a-3p inhibitor (Fig. 4A-K). These results suggested that TRAF1 could attenuate the inhibitory effect of miR-378a-3p on HCC cells.

### miR-378a-3p inhibited the activation of NF-κB signaling pathway by downregulating TRAF1 expression

**Fig. 5:**
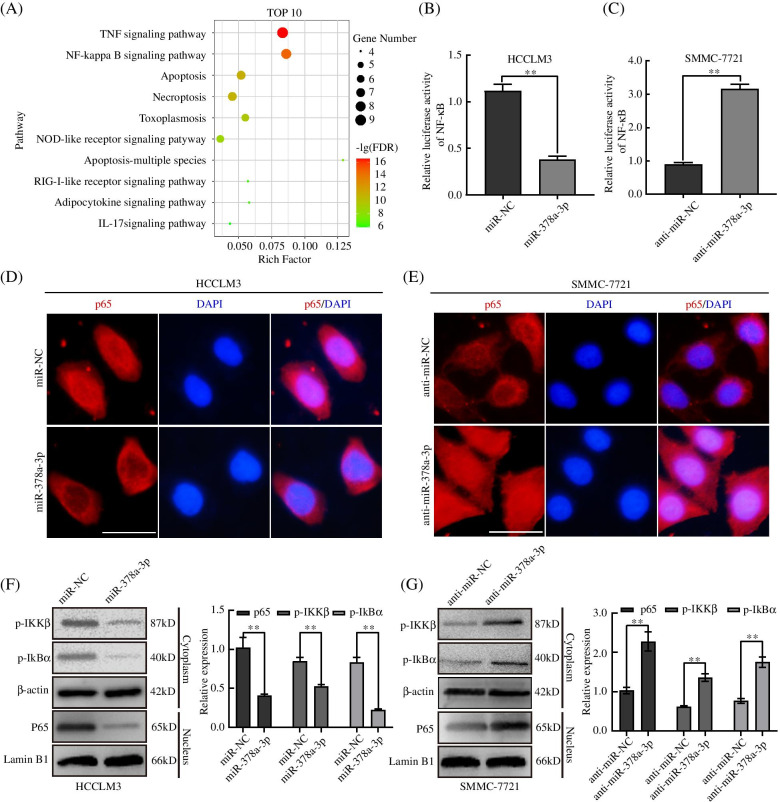
miR-378a-3p inhibited the activation of the NF-κB signaling pathway by downregulating TRAF1 expression. **A** Signaling pathway enrichment analysis of TRAF1 was performed using the online database STRING. **B, C** The relative luciferase activity of HCCLM3 and SMMC-7721 cells transfected with p65 and miR-378a-3p mimic or inhibitor was detected. **D, E** Immunofluorescence analysis was performed to detect NF-κB signaling activation in HCCLM3 and SMMC-7721 cells. **F, G** The protein expression of NF-κB target genes transfected with mimic, mimic-NC, inhibitor, or inhibitor-NC RNA was detected by western blotting. *P < 0.05; **P < 0.01; ***P < 0.001

To identify the molecular mechanism by which miR-378a-3p inhibits HCC angiogenesis. The STRING dataset was used to predict the top 10 functional pathways correlated with TRAF1 (Fig. 5A). As others have shown that, TRAF1 could activate the NF-κB signaling pathway and promotes cancer development [35]. Therefore, we performed luciferase reporter assays to determine the function of miR-378a-3p in NF-κB signaling activation and found that the relative luciferase activity of NF-κB was reduced in miR-378a-3p-overexpressing cells and was increased in miR-378a-3p knockdown cells (Fig. 5B, C). Moreover, the results of immunofluorescence assays showed that the nuclear translocation of p65 was decreased in miR-378a-3p-overexpressing cells but increased in miR-378a-3p knockdown cells (Fig. 5D, E). The western blot results showed that the overexpression of miR-378a-3p inhibited the phosphorylation of IκBα and IKKβ while miR-378a-3p knockdown increased their phosphorylation. Additionally, the upregulated expression of miR-378a-3p reduced nuclear p65 expression, whereas knockdown of miR-378a-3p increased nuclear p65 expression (Fig. 5F, G). To further characterize that NF-κB activation involved miR-378a-3p-mediated in HCC angiogenesis, we compared the cells transfected with miR-378a-3p NC, mimic, and mimic combined with LPS, the activator of NF-κB pathway, LPS reversed the proliferation, migration, invasion and angiogenesis abilities inhibitions of miR-378a-3p and the results were also confirmed by the transfection of miR-378a-3p inhibitor and SN50, the inhibitor of NF-κB pathway (Fig. S2A-J). ELISA also demonstrated that the secretion of VEGF in conditioned medium of HCC cell lines was upregulated by miR-378a-3p mimic and LPS compared with miR-378a-3p mimic alone, while the cells transfected with miR-378a-3p inhibitor and treated with SN50 exhibited lower secretion of VEGF (Fig. S2K). Moreover, western blot analysis confirmed that LPS attenuated the inhibitory effect of the miR-378a-3p mimic and that SN50 repressed the promoting effect of the miR-378a-3p inhibitor (Fig. S3A, B). Taken together, our data suggested that miR-378a-3p inhibited HCC angiogenesis by suppressing the activation of the NF-κB signaling pathway.

### miR-378a-3p is hypermethylated by DNMT1 and silenced in HCC tissues and cells

**Fig. 6:**
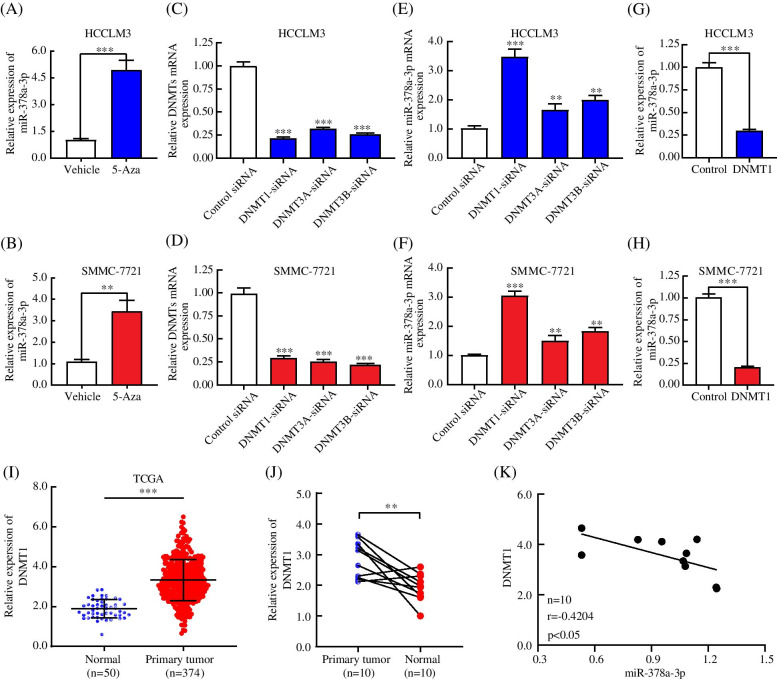
miR-378a-3p is hypermethylated by DNMT1 and silenced in HCC tissues and cells. **A, B** miR-378a-3p mRNA expression was detected in HCCLM3 and SMMC-7721 cells treated with 10 μM 5-Aza-dC for 48 h. **C, D** HCCLM3 and SMMC-7721 cells were transfected with DNMTs siRNA for 48 h, DNMTs mRNA expression was measured by qRT-PCR. **E, F** HCCLM3 and SMMC-7721 cells were transfected with DNMTs siRNA for 48 h, and miR-378a-3p mRNA expression was measured. **G, H** miR-378a-3p mRNA expression was detected in HCC cells transfected with pcDNA-DNMT1 and pcDNA-NC. **I, J** The mRNA expression of DNMT1 in the TCGA database and in 10 paired HCC and adjacent normal tissues is shown. **K** The relationship between DNMT1 and miR-378a-3p in 10 matched fresh HCC samples is shown. *P < 0.05; **P < 0.01; ***P < 0.001

Methylation of promoter DNA can reduce miRNA expression [36, 37]. We treated HCCLM3 and SMMC-7721 cells with a demethylating agent, 5-aza-CdR, to confirm whether the decrease in miR-378a-3p in HCC was caused by DNA methylation. As expected, the expression of miR-378a-3p was dramatically increased after treatment with 5-Aza-CdR (Fig. 6A, B). Because the miR-378a-3p promoter is hypermethylated in HCC, we hypothesized that the deregulation of a specific methylase or demethylase induces this process. To clarify the potential roles of the various DNMTs in mediating miR-378a-3p promoter methylation in HCC, we knocked down DNMT1, DNA methyltransferase 3 alpha (DNMT3A), and DNA methyltransferase 3 beta (DNMT3B) in HCC cells using specific small interfering RNAs (siRNAs) (Fig. 6C, D). The relative miR-378a-3p mRNA level of HCC cells transfected with DNMT1 siRNA, but not those transfected with DNMT3A siRNA and DNMT3B siRNA was obviously increased (Fig. 6E, F). Moreover, overexpression of DNMT1 significantly suppressed miR-378a-3p expression (Fig. 6G, H). To further test this speculation, we evaluated the expression level of DNMT1 in HCC patients with the TCGA database (Fig. 6I). We also examined the expression level of DNMT1 in 10 pairs of clinical HCC and normal tissues. The results revealed that DNMT1 was generally expressed at a higher level in HCC tissues (Fig. 6J). Spearman’s rank correlation analysis revealed a negative correlation between DNMT1 and miR-378a-3p expression (Fig. 6K). The above results suggested that DNMT1 significantly increased the hypermethylation of the miR-378a-3p promoter and suppressed the expression of miR-378a-3p.

### p65 promoted DNMT1 transcription and induced miR-378a-3p silencing mediated by DNA hypermethylation

**Fig. 7:**
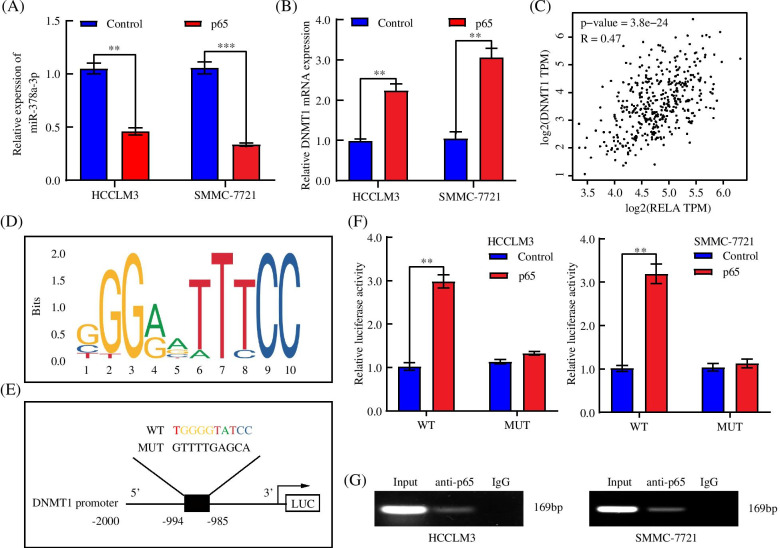
p65 promoted DNMT1 transcription and induced miR-378a-3p silencing mediated by DNA hypermethylation. **A** mRNA expression of miR-378a-3p was detected in different HCC cells transfected with control or p65. **B** The mRNA levels of DNMT1 were detected in HCC cells transfected with control or p65. **C** The relationship between DNMT1 and p65 in the GEPIA database. **D, E** Schematic diagram of binding sites from the JASPAR databases. **F, G** Detecting the function of p65 on the DNMT1 promoter by luciferase reporter and ChIP assays. *P < 0.05; **P < 0.01; ***P < 0.001

Consistent with our results, silencing miR-378a-3p can activate p65, which is a well-known transcription factor and is involved in tumorigenesis and development [38]. Interestingly, we found that p65 overexpression could downregulate the miR-378a-3p expression and upregulate DNMT1 mRNA expression in HCC cells (Fig. 7A, B). GEPIA database showed that p65 was positively correlated with DNMT1 in HCC (Fig. 7C). Based on this phenomenon, we assumed that p65 could transcription activate DNMT1, the methyltransferase of miR-378a-3p. To prove this assumption, we analyzed the DNMT1 promoter and identified potential binding sites for p65 (Fig. 7D) and then constructed vectors containing wild-type or mutant promoters of DNMT1 for a luciferase reporter assay (Fig. 7E). We found that the transfection of p65 significantly enhanced the luciferase activity of the DNMT1 WT reporter, whereas this effect was completely reversed by the mutant reporter, indicating that this site was the key region of p65 mediated DNMT1 upregulation (Fig. 7F). The results of the ChIP assay determined that p65 directly bound to the DNMT1 promoter in HCC cells (Fig. 7G). Taken together, these results suggested that DNMT1 could be activated by p65 and cause the epigenetic silencing of miR-378a-3p.

### The in vivo role of DNMT1/miR-378a-3p/TRAF1/p65 in regulating HCC angiogenesis

**Fig. 8:**
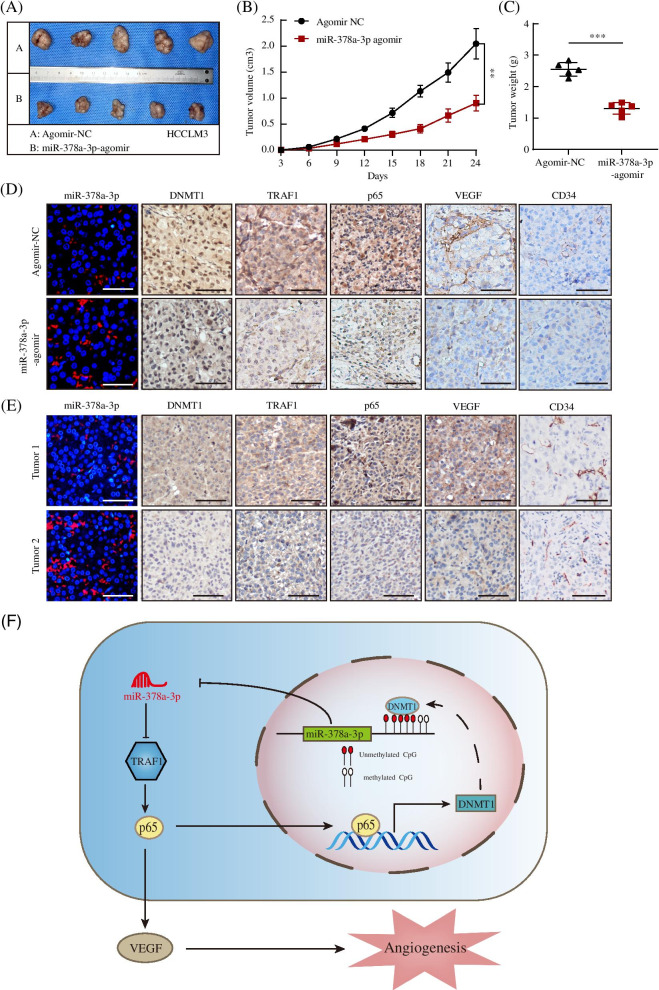
In vivo role of DNMT1/miR-378a-3p/TRAF1/p65 in regulating HCC angiogenesis. **A-C** Tumor growth of mice subcutaneously implanted with treated HCCLM3 cells. Tumor volume and weight were measured, and tumor size is shown. **D** Immunohistochemical staining of CD34, DNMT1, TRAF1, p65 and VEGF in the tumors from subcutaneously implanted mice. **E** FISH assay for miR-378a-3p and immunohistochemical staining for DNMT1, TRAF1, p65, VEGF and CD34 were performed on 10 human HCC specimens. **F** A working model showing how miR-378a-3p inhibits HCC tumor angiogenesis by inactivating the NF-κB signaling pathway. *P < 0.05; **P < 0.01; ***P < 0.001

Finally, we evaluated the effect of the DNMT1/miR-378a-3p/TRAF1/p65 axis on tumor growth and angiogenesis in vivo. The results showed that the miR-378a-3p agomir significantly inhibited tumor growth compared with the agomir-NC (Fig. 8A-C). IHC was carried out to detect DNMT1, TRAF1, p65, VEGF and CD34 in tumor tissues. The agomir-NC group showed higher expression of DNMT1, TRAF1, p65, VEGF and CD34 than miR-378a-3p agomir group (Fig. 8D). To investigate the clinical significance of the axis in promoting HCC angiogenesis, we examined the expression of miR-378a-3p, DNMT1, TRAF1, p65, VEGF and CD34 in HCC tissues and determined their relevance in HCC. The expression of miR-378a-3p was negatively correlated with the expression of DNMT1, TRAF1, p65, VEGF and CD34 in 10 matched fresh HCC samples (Fig. 8E, Fig. S4A-C). Moreover, we analyzed the correlation of miR-378a-3p, DNMT1, TRAF1 and VEGFA in TCGA data and found that the expression of miR-378a-3p was significantly inversely correlated with that of DNMT1, TRAF1, and VEGFA in HCC (Fig. S4D-F). Furthermore, we found that patients with HCC with high DNMT1, TRAF1 or VEGF mRNA levels had poorer survival rate via TCGA and SurvExpress databases (Fig. S4G-I). These data suggest that the DNMT1/miR-378a-3p/TRAF1/p65 axis inhibits HCC progression partly through restraining angiogenesis.

## Discussion

Angiogenesis plays pivotal roles in tumor progression and metastasis. Thus, it is important to investigate the role of miR-378a-3p in regulating HCC angiogenesis to develop therapeutic interventions. In this study, we found that the expression of miR-378a-3p was downregulated in HCC and was related to a poor prognosis. The expression of miR-378a-3p exerted a significant effect on angiogenesis according to a series of experiments in vitro and in vivo. Mechanistically, we also proved a positive feedback loop towards the hypermethylation of miR-378a-3p promoted by p65-mediated upregulation of DNMT1. A working model of our study has been summarized (Fig. 8F). Thus, we confirmed a new role for miR-378a-3p plays as an antiangiogenic factor in HCC, which provides a promising means for HCC treatment.

TRAF1, a member of the TRAF family, is known to regulate the cascade of canonical and non-canonical NF-κB signaling. Compared with the other members, TRAF1 has received more attention due to its lack of RING and zinc finger structures [39]. The upregulated antiapoptotic protein TRAF1 activates the PI3K/Akt/NF-κB signaling pathway in non-small cell lung cancer (NSCLC) [40]. TRAF1 is necessary for the development of UV radiation-induced skin cancer, and the deletion of TRAF1 in mice has been shown to significantly inhibit the formation of skin tumor [41]. Additionally, miR-483 has been shown to inhibit the proliferation and migration of colorectal cancer cells by targeting TRAF1 [42]. Studies have shown that miR-127-5p alleviates severe pneumonia by targeting TRAF1 to inactivate the Akt and NF-κB signaling pathways [43]. In our study, TRAF1 was confirmed as the downstream regulator of miR-378a-3p. Additionally, miR-378a-3p could negatively regulate the expression of TRAF1. These data further suggested that miR-378a-3p is inversely correlated with TRAF1 in HCC.

DNA methylation is one of the most important epigenetic modifications and is involved in various human diseases [44]. DNA methyltransferases (DNMTs) are a vital epigenetic family of enzymes that catalyze and maintain DNA methylation. Studies have identified three types of DNMTs with specific biological functions: DNMT1, DNMT3A, and DNMT3B. Compared with DNMT3A and DNMT3B, DNMT1 plays a more vital biological role [45]. Numerous studies have found that DNMT1 is associated with abnormal DNA methylation, and elevated expression of DNMT1 has been shown to promote the occurrence and development of cancers of the esophagus, breast, pancreas, thyroid, and colon [46]. Enhancer of Zeste homologue 2 (EZH2) has been shown to recruit DNMT1 to methylate the CpG island of the miR-484 promoter, which negatively regulates the Wnt/MAPK and TNF signaling pathways by upregulating the expression of HNF1A and MMP14, respectively, to inhibit the growth and metastasis of cervical cancer [47]. Jiang et al. found that ARID2 inhibited the epithelial-mesenchymal transition (EMT) of HCC cells by recruiting DNMT1 to the Snail promoter region, inducing promoter methylation and inhibiting Snail transcription [48]. Our results reveal a new mechanism indicating DNMT1-induced regulation of the downregulated expression of miR-378a-3p.

The expression of DNMT1 is regulated by multiple factors. Ubiquitinated histone H3 peptide (H3Ub) stimulates the maintenance activity of DNMT1 by increasing its methylation processivity. The SET and RING finger-associated domain (SRA) domains of ubiquitin-like with PHD and ring finger domain 1 (Uhrf1) additively stimulate DNMT1 activity with H3Ub peptides [49]. Deletion of lysine-specific histone demethylase 1 (LSD1) induces the loss of DNA methylation. This loss correlates with a decrease in DNMT1 protein expression, as a result of reduced DNMT1 stability [50]. Multiple transcription factors, including E2F1 and SP1, mediate the transcriptional activation of DNMT1 by the activating the MEK/ERK pathway [51]. In lung cancer, p65 is known to directly recruit DNMT1 to chromatin to enhance the methylation of the BRMS1 promoter; thus, acting as a transcriptional suppressor [52]. Therefore, we hypothesized that p65 induced DNMT1 transcription by promoting the activation of the promoter of DNMT1 in HCC. These results suggested that DNA promoter methylation might be a common approach of miR-378a-3p silencing in HCC. Downregulated expression of miR-378a-3p could promote TRAF1 expression, which leads to the upregulation of NF-κB signaling, thereby promoting a series of angiogenic effects in HCC cells. Interestingly, activated NF-κB could then in turn up-regulate DNMT1 expression, which forms a positive feedback loop that increased the methylation level of the miR-378a-3p promoter, thereby enhancing the angiogenesis effect of miR-378a-3p silencing and TRAF1 upregulation.

## Conclusion

In conclusion, our study revealed that miR-378a-3p is a potential predictor and therapeutic target for the treatment of HCC. Low miR-378a-3p expression was found to be associated with higher MVD and poor survival outcome in HCC patients. We found that DNA hypermethylation-induced silencing of miR-378a-3p facilitates HCC angiogenesis by upregulating TRAF1 and NF-κB signaling. As a transcriptional coactivator, p65 promotes DNMT1 expression, which positively regulates miR-378a-3p promoter methylation.

## Supplementary Information

**Additional file 1 **: **Table S1.** Sequences of siRNAs.

**Additional file 2 **: **Table S2.** Sequences of primers used for qRT-PCR and ChIP.

**Additional file 3 **: **Fig. S1.** miR-378a-3p expression inhibited HCC angiogenesis in vitro and in vivo. **(A)** MHCC-97H and MHCC-97 L cells were transfected with miR-378a-3p mimic, mimic-NC, inhibitor, or inhibitor-NC and analyzed by qRT-PCR. **(B-F)** Treated HUVECs were evaluated by the CCK-8 and colony formation assays to analyze cell viability. **(G-M)** Wound healing, invasion and tube formation assays were performed to analyze angiogenesis. **(N)** The VEGF protein concentration in conditioned medium of treated MHCC-97H and MHCC-97 L cells was detected by ELISA. *P < 0.05; **P < 0.01; ***P < 0.001.

**Additional file 4 **: **Fig. S2.** miR-378a-3p suppressed endothelial cell proliferation, migration, invasion and angiogenesis by directly regulating NF-κB. **(A-D)** HUVECs were co-cultured with treated HCCLM3 and SMMC-7721 cells for 48 h. Treated HUVECs were evaluated by the CCK-8 and colony formation assays to analyze cell viability. **(E-H)** Wound healing and invasion assays were performed. **(I, J)** HUVEC tube formation assays were performed and cultured with conditioned supernatant collected from HCC cells. **(K)** The VEGF protein concentration in the conditioned medium of treated HCC cells was detected by ELISA. *P < 0.05; **P < 0.01; ***P < 0.001.

**Additional file 5 **: **Fig. S3.** miR-378a-3p suppressed tumor progression by directly regulating NF-κB. **(A, B)** The protein levels of TRAF1 and NF-κB were detected in HCCLM3 and SMMC-7721 cells treated with miR-NC, miR-378a-3p, LPS, and anti-miR-NC, anti-miR-378a-3p, and SN50. *P < 0.05; **P < 0.01; ***P < 0.001.

**Additional file 6 **: **Fig. S4.** In vivo role of DNMT1/miR-378a-3p/TRAF1/p65 in regulating HCC angiogenesis. **(A, B, C)** Correlation analysis of miR-378a-3p and DNMT1, TRAF1 and VEGFA 10 human HCC specimens. **(D, E, F)** Correlation analysis of miR-378a-3p and DNMT1, TRAF1 and VEGFA in HCC with data from the TCGA dataset. **(G, H, I)** OS analysis according to DNMT1 and VEGFA expression in patients with HCC in the TCGA dataset. *P < 0.05; **P < 0.01; ***P < 0.001.

## Data Availability

The datasets used or analysed during the current study are available from the corresponding author on reasonable request.

## Abbreviations

HCC: Hepatocellular carcinoma
CCK-8: Cell Counting Kit-8
ChIP: Chromatin immunoprecipitation
FISH: Fluorescence in situ hybridization
MVD: Microvascular density
HUVECs: Human umbilical vein endothelial cells
TRAF1: TNF receptor associated factor 1
VEGF: Vascular endothelial growth factor
DNMT1: DNA methyltransferase 1
REC8: Meiotic recombination protein
PDSS2-DEL2: Prenyl diphosphate synthase subunit 2
MicroRNAs: MiRNAs
KLK4: Kallikrein-related peptidase 4
MAP K1: Mitogen-activated protein kinase 1
GRB2: Growth factor receptor bound protein 2
FBS: Fetal bovine serum
NSCLC: Non-small cell lung cancer
qRT-PCR: Quantitative reverse; transcriptase polymerase chain reaction
GEO: Gene Expression Omnibus
TCGA: The Cancer Genome Atlas
GEPIA: Gene Expression Profiling Interactive Analysis
STRING: Search Tool for the Retrieval of Interacting Genes/Proteins
RNA-seq: RNA sequencing
OS: Overall survival
DFS: Disease-free survival
EZH2: Enhancer of Zeste Homolog 2
UTR: Untranslated region
DEGs: Differentially expressed genes
LPS: Lipopolysaccharide
DNMT3A: DNA methyltransferase 3 alpha
DNMT3B: DNA methyltransferase 3 beta
siRNAs: Small interfering RNAs
EMT: Epithelial-mesenchymal transition
H3Ub: H3 peptide ubiquitinated
SRA: SET and RING finger-associated
Uhrf1: Ubiquitin like with PHD and ring finger domains 1
LSD1: Lysine-specific histone demethylase 1.
