# Supplementary material for: DNMT1-induced miR-378a-3p silencing promotes angiogenesis via the NF-κB signaling pathway by targeting TRAF1 in hepatocellular carcinoma
Source: J Exp Clin Cancer Res. 2021 Nov 8;40:352. doi: 10.1186/s13046-021-02110-6 (PMC8576931; doi:10.1186/s13046-021-02110-6)
Supplement: Supplementary file 1 — Additional file 1 : Table S1. Sequences of siRNAs. [file 13046_2021_2110_MOESM1_ESM.docx]

**Table S1. siRNA sequences used in this study**

| Gene | siRNA Sequence |
| --- | --- |
| Control siRNA | UUCUCCGAACGUGUCACGUTT |
| DNMT1 siRNA | CAATGAGACTGACATCAAA |
| DNMT3A siRNA | GCCTGGAGCCACCAGAAGA |
| DNMT3B siRNA | GCAACGATCTCTCAAATGT |
| TRAF1 siRNA | AAACCCAGGGCTGCCTTGGAAAAG |
