# Supplementary material for: DNMT1-induced miR-378a-3p silencing promotes angiogenesis via the NF-κB signaling pathway by targeting TRAF1 in hepatocellular carcinoma
Source: J Exp Clin Cancer Res. 2021 Nov 8;40:352. doi: 10.1186/s13046-021-02110-6 (PMC8576931; doi:10.1186/s13046-021-02110-6)
Supplement: Supplementary file 2 — Additional file 2 : Table S2. Sequences of primers used for qRT-PCR and ChIP. [file 13046_2021_2110_MOESM2_ESM.docx]

| **Table S2. Primer sequences used in real-time RT-PCR and ChIP** | |
| --- | --- |
| Real time RT-PCR | Primer Sequence (5’-3’) |
| TRAF1 | F: CTTGGAGCAGAGGGTGGT |
|  | R: GCCTGGTGACATTGGTGAT |
| DNMT1 | F: CCTAGCCCCAGGATTACAAGG |
|  | R: ACTCATCCGATTTGGCTCTTTC |
| DNMT3A | F: TGTAACGAAGTGAAGGAGGAGAA |
|  | R: CATCTTGCCGAGGGAGTCT |
| DNMT3B | F: AGGGAAGACTCGATCCTCGTC |
|  | R: GTGTGTAGCTTAGCAGACTGG |
| GAPDH | F: TGCACCACAACTGCTTAGC |
|  | R: GGCATGGACTGTGGTCATGAG |
| U6 | F: ATTGGAACGATACAGAGAAGATT |
|  | R: GGAACGCTTCACGAATTTG |
|  |  |
| ChIP | F: TGTCACCATGCCCAGCAAAT |
|  | R: TAAATTAAGAAGCACCATGT |
